# Catheter ablation of ventricular tachycardia in patients with arrhythmogenic right ventricular cardiomyopathy and biventricular involvement

**DOI:** 10.1093/europace/euae059

**Published:** 2024-02-28

**Authors:** Lishui Shen, Shangyu Liu, Zhenhao Zhang, Yulong Xiong, Zihao Lai, Feng Hu, Lihui Zheng, Yan Yao

**Affiliations:** Arrhythmia Center, National Center for Cardiovascular Diseases, Chinese Academy of Medical Sciences and Peking Union Medical College, Fuwai Hospital, No. 167 North Lishi Road, Xicheng District, Beijing 100037, China; Department of Cardiology, Shanghai Tenth People’s Hospital, Tongji University, Shanghai 200072, China; Arrhythmia Center, National Center for Cardiovascular Diseases, Chinese Academy of Medical Sciences and Peking Union Medical College, Fuwai Hospital, No. 167 North Lishi Road, Xicheng District, Beijing 100037, China; Department of Cardiology, The First Hospital of Hebei Medical University, Shijiazhuang 050031, China; Arrhythmia Center, National Center for Cardiovascular Diseases, Chinese Academy of Medical Sciences and Peking Union Medical College, Fuwai Hospital, No. 167 North Lishi Road, Xicheng District, Beijing 100037, China; Arrhythmia Center, National Center for Cardiovascular Diseases, Chinese Academy of Medical Sciences and Peking Union Medical College, Fuwai Hospital, No. 167 North Lishi Road, Xicheng District, Beijing 100037, China; Arrhythmia Center, National Center for Cardiovascular Diseases, Chinese Academy of Medical Sciences and Peking Union Medical College, Fuwai Hospital, No. 167 North Lishi Road, Xicheng District, Beijing 100037, China; Department of Cardiology, Renji Hospital, School of Medicine, Shanghai Jiaotong University, Shanghai 200127, China; Arrhythmia Center, National Center for Cardiovascular Diseases, Chinese Academy of Medical Sciences and Peking Union Medical College, Fuwai Hospital, No. 167 North Lishi Road, Xicheng District, Beijing 100037, China; Arrhythmia Center, National Center for Cardiovascular Diseases, Chinese Academy of Medical Sciences and Peking Union Medical College, Fuwai Hospital, No. 167 North Lishi Road, Xicheng District, Beijing 100037, China

**Keywords:** Arrhythmogenic right ventricular cardiomyopathy, Biventricular involvement, Ventricular tachycardia, Catheter ablation

## Abstract

**Aims:**

Catheter ablation of ventricular tachycardia (VT) improves VT-free survival in ‘classic’ arrhythmogenic right ventricular cardiomyopathy (ARVC). This study aims to investigate electrophysiological features and ablation outcomes in patients with ARVC and biventricular (BiV) involvement.

**Methods and results:**

We assembled a retrospective cohort of definite ARVC cases with sustained VTs. Patients were divided into the BiV (BiV involvement) group and the right ventricular (RV) (isolated RV involvement) group based on the left ventricular systolic function detected by cardiac magnetic resonance. All patients underwent electrophysiological mapping and VT ablation. Acute complete success was non-inducibility of any sustained VT, and the primary endpoint was VT recurrence. Ninety-eight patients (36 ± 14 years; 87% male) were enrolled, including 50 in the BiV group and 48 in the RV group. Biventricular involvement was associated with faster clinical VTs, a higher VT inducibility, and more extensive arrhythmogenic substrates (all *P* < 0.05). Left-sided VTs were observed in 20% of the BiV group cases and correlated with significantly reduced left ventricular systolic function. Catheter ablation achieved similar acute efficacy between these two groups, whereas the presence of left-sided VTs increased acute ablation failure (40 vs. 5%, *P* = 0.012). Over 51 ± 34 months [median, 48 (22–83) months] of follow-up, cumulative VT-free survival was 52% in the BiV group and 58% in the RV group (*P* = 0.353). A multivariate analysis showed that younger age, lower RV ejection fraction (RVEF), and non-acute complete ablation success were associated with VT recurrence in the BiV group.

**Conclusion:**

Biventricular involvement implied a worse arrhythmic phenotype and increased the risk of left-sided VTs, while catheter ablation maintained its efficacy for VT control in this population. Younger age, lower RVEF, and non-acute complete success predicted VT recurrence after ablation.

## Graphical Abstract

**Graphical Abstract:**
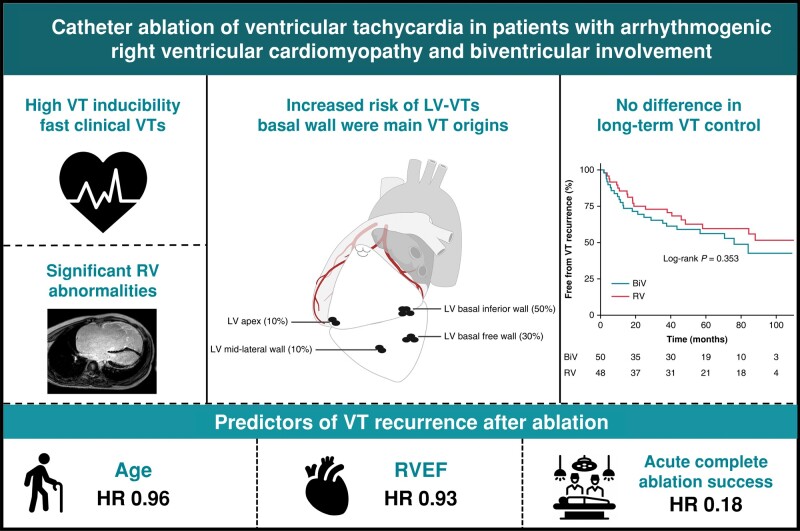

**What’s new?**

- Biventricular (BiV) involvement in arrhythmogenic right ventricular cardiomyopathy (ARVC) correlated with faster clinical ventricular tachycardias (VTs), a higher VT inducibility, and more extensive arrhythmogenic substrates.
- Left-sided VTs were not rare when the left ventricle was involved, and these VTs were more malignant than right-sided VTs and associated with significantly reduced left ventricular systolic function.
- Catheter ablation achieved similar acute and long-term efficacy for VT control between patients with and without BiV involvement, whereas the presence of left-sided VTs increased acute ablation failure.
- Younger age, lower right ventricular ejection fraction, and partial success or failure in the procedure were predictors of VT recurrence after ablation in patients with BiV involvement.

## Introduction

Arrhythmogenic right ventricular cardiomyopathy (ARVC) is an inherited cardiomyopathy characterized by loss of myocardium and fibrofatty infiltration.^1,2^ These peculiar pathological changes contribute to arrhythmogenic substrates, thereby increasing the risk of ventricular arrhythmias (VAs) and sudden cardiac death (SCD).^3^ Due to a lack of causative treatment for ARVC, the placement of an implantable cardioverter defibrillator (ICD) remains the cornerstone of SCD prevention in this setting. A recent study indicated that those patients without an ICD had an annualized SCD rate of 7.21 per 1000.^4^ Although a series of individual-tailored risk-stratification tools have improved the recognition of high-risk subjects, strategies for treating these malignant cases are still very limited.^5^ Catheter ablation has confirmed its value in reducing ventricular tachycardia (VT) burden and electrical storm recurrence in ARVC and is recommended by current guidelines and expert consensus.^6–9^ Notably, most previous ablation data were obtained from cases with isolated right ventricular (RV) involvement, known as the ‘classic’ ARVC. The phenotype of biventricular (BiV) involvement is not rare in ARVC,^10,11^ whereas electrophysiological features and ablation efficacy for VT control in this population remain unclear. In the present study, we report our institutional experience on catheter ablation of VT in patients with ARVC and BiV involvement and compare clinical characteristics and ablation outcomes between patients with and without BiV involvement.

## Methods

### Study population

**Figure 1:**
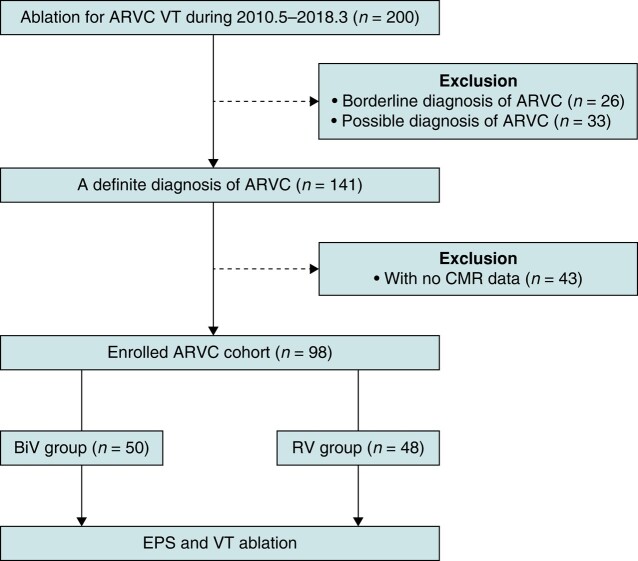
A flowchart of the study selection process. ARVC, arrhythmogenic right ventricular cardiomyopathy; BiV, biventricular; CMR, cardiac magnetic resonance; EPS, electrophysiological study; RV, right ventricular; VT, ventricular tachycardia.

From May 2010 to March 2018, consecutively selected ARVC patients who received VT ablation in our centre were included in the study (*Figure 1*). The inclusion criterion was a definite ARVC diagnosis according to the 2010 revised Task Force Criteria (two major criteria or one major criterion plus two minor criteria or four minor criteria).^12^ The exclusion criterion was the lack of cardiac magnetic resonance (CMR) data. All patients had at least one episode of symptomatic, sustained, monomorphic VT documented by using a 12-lead electrocardiogram (ECG) or Holter monitoring. The study conformed to the Declaration of Helsinki and was approved by the institutional ethics committee. All patients signed the informed consent form.

The study population was divided into two groups: the BiV group (BiV involvement) and the RV group (isolated RV involvement). Right ventricular involvement was considered if the patient met a definite ARVC diagnosis. Biventricular involvement was defined based on the CMR demonstration of left ventricular (LV) systolic dysfunction [an LV ejection fraction (LVEF) ≤48% for men and 50% for women].^13^ Baseline demographic data, ECG features, CMR data, and drug therapies were collected. Genetic testing was not part of the study protocol, but available results were considered for analysis.

### Electrophysiologic study and mapping

The electrophysiologic study (EPS) protocol was reported previously.^14,15^ Anti-arrhythmic drugs were discontinued at least five half-lives before EPS; amiodarone was discontinued ≥5 days before the procedure. All patients underwent the procedure under local anaesthesia and conscious sedation. Programmed stimulation, including ramp pacing with up to three ventricular extra stimuli to a minimum coupling interval of 200 ms and burst pacing with the shortest cycle length (CL) of 200 ms, was delivered from two different RV/LV sites to induce VTs. Intravenous isoproterenol infusion was administered (maximum dose, 6 μg/min) when necessary. Clinical VT was defined as the same 12-lead surface ECG morphology and similar CL. Other induced monomorphic VTs were considered as non-clinical VTs. For haemodynamically tolerated monomorphic sustained VTs, activation and entrainment mapping were performed to identify the critical sites associated with the initiation or maintenance of VT. If the VT was non-sustained, haemodynamically unstable, or not inducible, pace mapping was used to reproduce the clinical VT morphology and identify the location of the best pace mapping. Substrate mapping was performed to identify low-voltage areas and abnormal potentials. Low-voltage amplitude in the bipolar electrogram was defined as <1.5 mV for the endocardial map and <1.0 mV for the epicardial map.^16^ Abnormal potentials were defined as follows: delayed potentials, >10 ms after the end of the QRS complex; split potentials, an isoelectric period of >30–50 ms between spikes; fragmented potentials, multi-component electrograms, <0.5 mV and >133 ms.^17,18^

### Ablation strategy, endpoints, and complications

Radiofrequency ablation was performed at the site of pre-systolic potentials during VT in case of haemodynamically tolerated sustained VTs and short post-pacing intervals after entrainment of VT. Delayed, split, or fragmented potentials judged crucial to maintaining the induced VT circuits were also targeted for ablation. For VTs that were non-sustained, unstable, or not inducible, pace mapping–guided ablation, along with the elimination of all fractionated low-voltage signals/delayed/split potentials, was performed in the region where the documented clinical VTs were suspected. An endocardial approach was used initially, with epicardial ablation reserved for those who failed an endocardial ablation or lacked an endocardial substrate. The regional ablation strategy with an irrigated catheter under an output energy limit of 30–40 W and a maximum temperature limit of 45°C was applied for all patients. After ablation, if the only inducible VAs were polymorphic VT or ventricular fibrillation (VF), no further ablation was performed. The electrophysiological mapping and ablation were performed under the guidance of Ensite NavX (St. Jude Medical, St. Paul, MN, USA) or CARTO system (Biosense Webster, Irvine, CA, USA).

Acute complete success was defined as non-inducibility of any sustained VT at the end of the procedure. Partial success was defined as non-inducibility of clinical VTs but the remaining inducibility of non-clinical sustained VTs. In the case of unstable VT or VT non-inducibility during EPS, the procedural endpoint was the elimination of all fractionated low-voltage signals/delayed/split potentials in the region where the documented clinical VTs were suspected. Acute ablation failure was considered if clinical VTs remained inducible. Major procedural complications were pericardial tamponade, pericarditis, ventricle perforation, pneumothorax, pulmonary thromboembolism, or other vascular complications requiring surgical intervention.

### Follow-up and long-term outcomes

Patients were followed up every 6 months in the outpatient clinic or by telephone. A 12-lead surface ECG and a Holter test were performed routinely and at the time of symptoms. The primary outcome was VT recurrence, defined as any sustained VT/VF, including any appropriate ICD intervention, namely shock or anti-tachycardia pacing. The secondary outcome was the composite endpoint of all-cause death or heart transplantation.

### Statistics

Continuous variables were presented as mean ± standard deviation or median (inter-quartile range). Categorical variables were summarized as count and percentage. Comparison between groups was performed using the Student’s *t*-test or Mann–Whitney *U* test for continuous variables and the *χ*^2^ test or the Fisher’s exact test for categorical variables. Kaplan–Meier survival analyses and log-rank tests were used to compare the long-term outcomes between groups. The effect of different clinical variables on VT-free survival in the BiV group was investigated using the Cox proportional hazards model. The variables associated with VT recurrence were screened by a univariate analysis, and those with a *P*-value <0.10 were further analysed by a multivariate analysis. A two-tailed *P*-value <0.05 was considered significant. All statistical analyses were performed using SPSS 22.0 software (SPSS Inc., Chicago, IL, USA).

## Results

### Patient characteristics

Ninety-eight patients were enrolled. The clinical characteristics of the study population are shown in *Table 1*. The mean age was 36 ± 14 years, and most patients were male (85/98, 87%). Fifty patients had BiV involvement, and 48 had isolated RV involvement. Patients in the BiV group had a higher proportion of syncope (44 vs. 25%; *P* = 0.048) and heart failure (28 vs. 10%; *P* = 0.028) symptoms, a larger RV end-diastolic volume index (136 ± 26 vs. 118 ± 17 mL/m^2^; *P* = 0.009), and a lower RV ejection fraction (RVEF, 26 ± 6 vs. 32 ± 8%; *P* < 0.001) when compared with those in the RV group (see Supplementary material online, *Figure S1* for the ECG and CMR findings of a representative patient with ARVC and BiV involvement). In addition, the BiV group patients showed a larger LV end-diastolic volume index and a higher prevalence of intramyocardial fat infiltration and late gadolinium enhancement in the LV (all *P* < 0.001). Eight patients in the BiV group and nine in the RV group implanted an ICD before VT ablation. The genetic background between two groups showed no significant difference, and the Plakophilin-2 (*PKP2*) mutation was the most commonly seen genotype in both groups (see Supplementary material online, *Table S1* for the detailed genetic information).

**Table 1:** Demographic and clinical characteristics of the study population according to the ventricular involvement group

	Total population (*N* = 98)	BiV group (*N* = 50)	RV group (*N* = 48)	*P*-value*
Male	85 (87)	42 (84)	43 (90)	0.415
Age at baseline, years	36 ± 14	40 ± 14	32 ± 14	0.011
Syncope	34 (35)	22 (44)	12 (25)	0.048
Heart failure	19 (19)	14 (28)	5 (10)	0.028
Aborted cardiac arrest	28 (29)	15 (30)	13 (27)	0.749
ICD	17 (17)	8 (16)	9 (19)	0.719
Family history	36 (37)	16 (32)	20 (42)	0.321
Performed genetic test	86 (88)	45 (90)	41 (85)	0.695
Desmosome	45 (52)	21 (47)	24 (59)	0.271
Plakophilin-2	33 (38)	15 (33)	18 (44)	0.314
Desmoglein-2	9 (10)	5 (11)	4 (10)	>0.999
Desmocollin-2	4 (5)	3 (7)	1 (2)	0.618
Desmoplakin	4 (5)	2 (4)	2 (5)	>0.999
Non-desmosome	9 (10)	6 (13)	3 (7)	0.488
None mutation	32 (37)	18 (40)	14 (34)	0.575
CMR features				
RV-EDVI, mL/m^2^	126 ± 23	136 ± 26	118 ± 17	0.009
RVEF, %	29 ± 8	26 ± 6	32 ± 8	<0.001
RV fat infiltration	57 (58)	29 (58)	28 (58)	0.973
RV-LGE	92 (94)	48 (96)	44 (92)	0.636
LV-EDVI, mL/m^2^	87 ± 24	96 ± 29	78 ± 12	<0.001
LVEF, %	50 ± 11	41 ± 8	59 ± 5	<0.001
LV fat infiltration	27 (28)	22 (44)	5 (10)	<0.001
LV-LGE	54 (55)	46 (92)	8 (17)	<0.001
AADs at baseline				
Beta-blocker	23 (23)	14 (28)	9 (19)	0.280
Sotalol	63 (64)	30 (60)	33 (69)	0.366
Amiodarone	7 (7)	4 (8)	3 (6)	0.955
Class 1	26 (27)	12 (24)	14 (29)	0.563
AADs at last follow-up				
Beta-blocker	32 (33)	19 (38)	13 (27)	0.249
Sotalol	33 (34)	14 (28)	19 (40)	0.225
Amiodarone	5 (5)	3 (6)	2 (4)	0.963
Class 1	20 (20)	9 (18)	11 (23)	0.546

Variables are expressed as number of patients (%) or mean ± standard deviation.

AADs, anti-arrhythmic drugs; BiV, biventricular; CMR, cardiovascular magnetic resonance; EDVI, end-diastolic volume index; ICD, implantable cardioverter defibrillator; LGE, late gadolinium enhancement; LV, left ventricular; LVEF, LV ejection fraction; RV, right ventricular; RVEF, RV ejection fraction.

^*^ *P*-value for the BiV group vs. the RV group.

### Baseline electrocardiographic findings

The main baseline ECG findings are reported in *Table 2*. An epsilon wave presented in 18 patients. Seventy-three patients (73/98, 74%) had T-wave inversion (TWI) in the right precordial leads (V1–V3). The BiV group patients exhibited more frequent TWI in leads V1–V6 (48 vs. 15%; *P* < 0.001) and low QRS voltages in limb leads (44 vs. 13%; *P* = 0.001, see Supplementary material online, *Figure S2* for the representative examples of ECGs in patients with BiV involvement and isolated RV involvement). There were 18 patients in the BiV group with coexisting TWI in leads V1–V6 and low QRS voltages in limb leads, which was six-fold of that in the RV group. The ECG documented clinical VTs with a left bundle branch block (LBBB) morphology in 48 patients and a right bundle branch block (RBBB) morphology in 10 patients in the BiV group. In the RV group, only LBBB-like VTs were recorded. The CL of clinical VTs in the BiV group was significantly shorter than that in the RV group (305 ± 73 vs. 342 ± 70 ms; *P* = 0.036). In the BiV group, LBBB-like VTs were significantly faster than RBBB-like VTs (278 ± 33 vs. 327 ± 52 ms; *P* = 0.025) and were associated with reduced LVEF (35 ± 9 vs. 43 ± 10%; *P* = 0.018).

**Table 2:** Electrocardiographic characteristics of the study population according to the ventricular involvement group

	Total population (*N* = 98)	BiV group (*N* = 50)	RV group (*N* = 48)	*P*-value*
Heart rate, b.p.m.	70 ± 11	70 ± 11	70 ± 12	0.946
PR interval, ms	164 ± 30	171 ± 29	157 ± 29	0.023
QRS duration, ms	106 ± 26	110 ± 25	103 ± 27	0.572
QTc interval, ms	432 ± 42	434 ± 50	429 ± 32	0.194
Epsilon waves	18 (18)	11 (22)	7 (15)	0.343
TWI in V1–V3	73 (74)	39 (78)	34 (71)	0.416
TWI in V1–V6	31 (32)	24 (48)	7 (15)	<0.001
Low QRS voltages in limb leads	28 (29)	22 (44)	6 (13)	0.001
TWI in V1–V6 and low QRS voltages in limb leads	21 (21)	18 (36)	3 (6)	<0.001
Cycle length of clinical VTs, ms	322 ± 73	305 ± 73	342 ± 70	0.036
Clinical VT morphology				
LBBB-like	96 (98)	48 (96)	48 (100)	0.495
RBBB-like	10 (10)	10 (20)	0	0.001

Variables are expressed as number of patients (%) or mean ± standard deviation.

BiV, biventricular; LBBB, left bundle branch block; RBBB, right bundle branch block; RV, right ventricular; TWI, T-wave inversion; VT, ventricular tachycardia.

^*^ *P*-value for the BiV group vs. the RV group.

### Electrophysiological study and acute ablation outcomes

**Figure 2:**
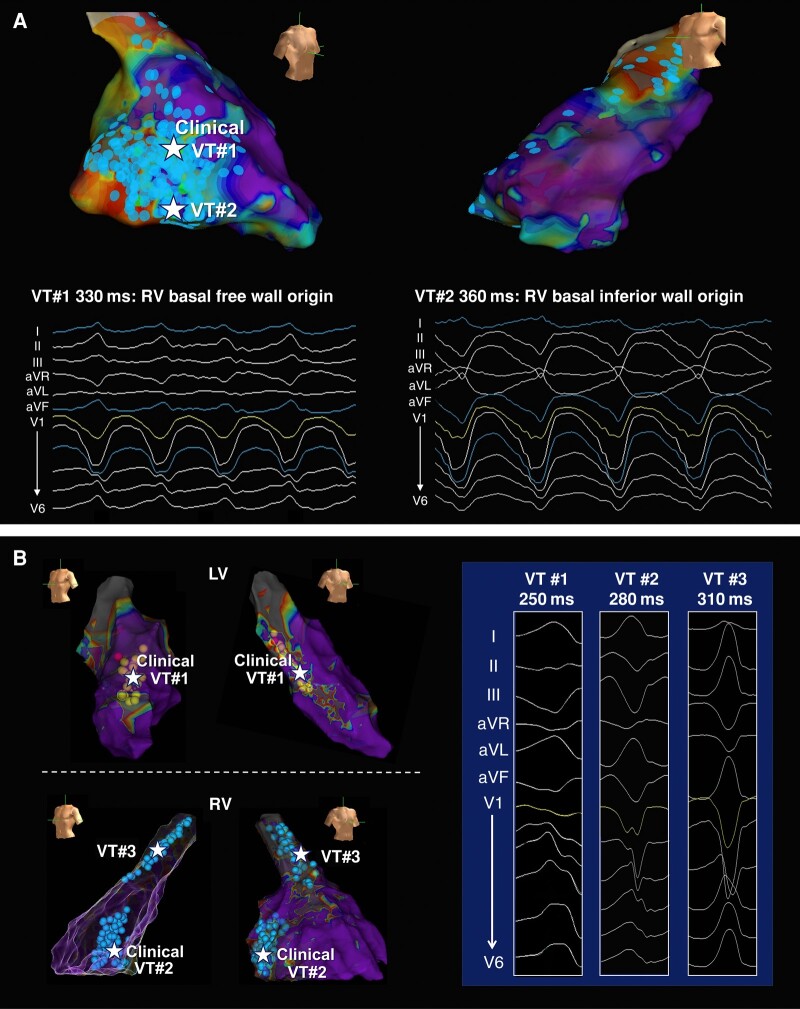
Examples of catheter ablation in patients with arrhythmogenic RV cardiomyopathy. (*A*) A representative case with isolated RV involvement and two distinct inducible VT morphologies. Extensive substrate ablation targeting both mappable VT and abnormal potentials rendered VT non-inducible with programmed stimulation from 2 different RV sites using ≤3 extra stimuli. (*B*) A representative case with biventricular involvement and three distinct inducible VT morphologies. Two clinical VTs originated from the RV outflow tract and the LV inferior wall (submitral area), respectively. Another induced VT arose from the RV free wall (subtricuspid region). At the end of ablation, VT was non-inducible with programmed stimulation from 2 different RV sites using ≤3 extra stimuli. LV, left ventricular; RV, right ventricular; VT, ventricular tachycardia.

**Figure 3:**
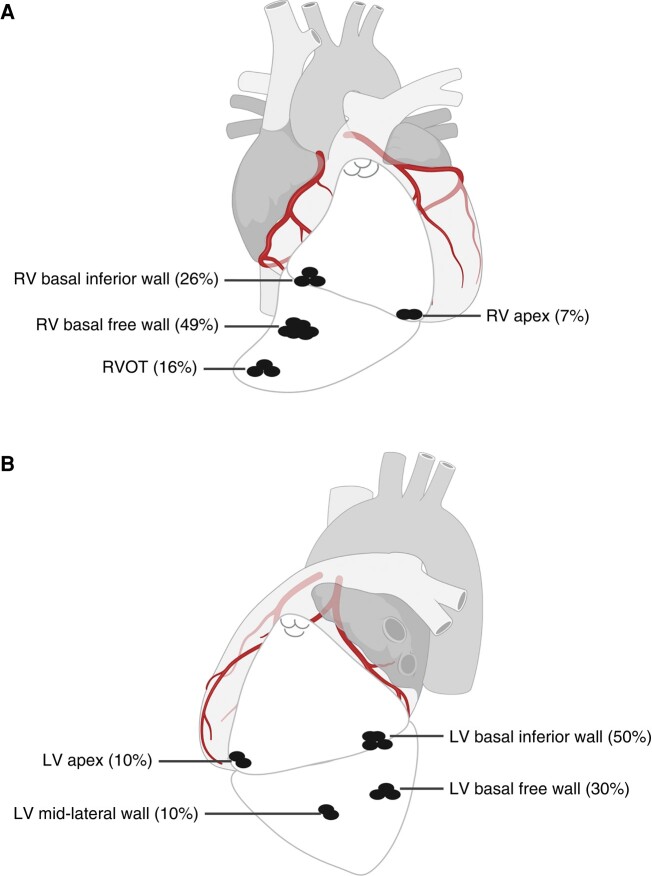
The distribution of critical sites of VT in patients with arrhythmogenic RV cardiomyopathy and biventricular involvement. (*A*) Critical sites distribution of right-sided VT. (*B*) Critical sites distribution of left-sided VT. LV, left ventricular; RV, right ventricular; RVOT, RV outflow tract; VT, ventricular tachycardia.

Detailed procedural data are given in *Table 3*. Ventricular tachycardia was induced in 78 patients with a median of 2 types [range (1–6)] per patient (*Figure 2A* and *B*). The BiV group showed a significantly higher VT inducibility than the RV group (90 vs. 69%; *P* = 0.009). Forty-three patients (43/50, 86%) in the BiV group induced RV-VTs, and the CL was shorter than that in the RV group (302 ± 49 vs. 330 ± 49 ms; *P* = 0.017). In addition, the BiV group showed a larger RV endocardial bipolar low-voltage area (51.2 ± 26.8 vs. 23.6 ± 17.9 cm^2^; *P* < 0.001). There were no statistical differences between these two groups regarding the distribution of critical sites of RV-VTs. Right ventricular basal free wall, basal inferior wall, and RV outflow tract (RVOT) were the main VT origin sites in the BiV group (*Figure 3A*).

**Table 3:** Comparison of procedural data between two groups

	BiV (*N* = 50)	RV (*N* = 48)	*P-*value
VT inducibility	45 (90)	33 (69)	0.009
No. of induced VT types			
1	23 (46)	13 (27)	0.052
2	7 (14)	8 (17)	0.714
≥3	15 (30)	12 (25)	0.580
Only PVCs	5 (10)	15 (31)	0.009
Induced RV-VTs	43 (86)	33 (69)	0.041
Cycle length, ms	302 ± 49	330 ± 49	0.017
Critical sites			
RVOT	7 (16)	9 (27)	0.244
RV basal free wall	21 (49)	19 (58)	0.450
RV basal inferior wall	11 (26)	11 (33)	0.460
RV apex	3 (7)	5 (15)	0.439
Induced LV-VTs	10 (20)	0	0.001
Cycle length, ms	268 ± 29	NA	
Critical sites			
LV basal inferior wall	5 (50)	NA	
LV basal free wall	3 (30)	NA	
LV apex	1 (10)	NA	
Acute efficacy			
Complete success	33 (66)	35 (73)	0.458
Partial success	11 (22)	9 (19)	0.690
No success	6 (12)	4 (8)	0.549

Variables are expressed as number of patients (%) or mean ± standard deviation.

BiV, biventricular; LV, left ventricular; NA, not applicable; PVC, premature ventricular contraction; RV, right ventricular; RVOT, RV outflow tract; VT, ventricular tachycardia.

No patient in the RV group induced LV-VTs, whereas 10 patients (10/50, 20%) in the BiV group induced left-sided sustained VTs. The mean number of induced LV-VTs was 1.5 ± 0.7 per patient. These LV-VTs showed a much shorter CL than RV-VTs (268 ± 29 vs. 302 ± 49 ms; *P* = 0.040). Five patients (5/10, 50%) had unstable haemodynamics or VF events during LV-VT episodes. Left ventricular basal inferior wall and LV basal free wall were the main origin sites of these LV-VTs (*Figure 3B*).

Radiofrequency ablation procedures were performed in all patients, with 92 receiving endocardial-alone procedures and 6 endo-epicardial combined ablation. After ablation, acute complete success was achieved in 33 patients (33/50, 66%) in the BiV group and 35 (35/48, 73%) in the RV group. Partial success was documented in 11 patients (11/50, 22%) in the BiV group and 9 (9/48, 19%) in the RV group. Six patients (6/50, 12%) in the BiV group and four (4/48, 8%) in the RV group had ablation failure (for the detailed procedural data, see Supplementary material online, *Table S2*). There was no difference in acute ablation efficacy between these two groups (all *P* > 0.05). The 10 cases with ablation failure inserted an ICD, including three patients in the BiV group and two patients in the RV group had an ICD before ablation.

**Figure 4:**
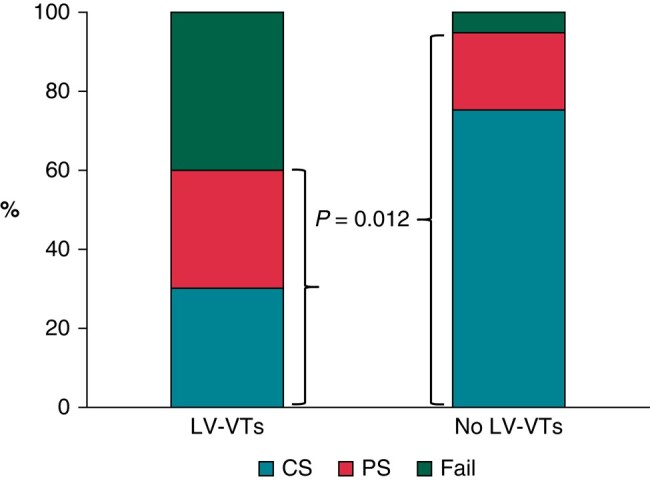
Difference in acute ablation efficacy between patients with and without left-sided VT in the group of biventricular involvement. Patients with LV-VTs showed a significantly lower ablation efficacy than those without LV-VTs. CS, complete success; Fail, ablation failure; LV-VTs, left ventricular-originated VTs; PS, partial success; VT, ventricular tachycardia.

Among 10 patients inducing LV-VTs in the BiV group, 3 patients received epicardial ablation due to the failure of the endocardial approach and 7 patients underwent endocardial-alone ablation (see Supplementary material online, *Table S3* for main characteristics). Finally, three patients achieved complete success, three achieved partial success, and the remaining four documented ablation failure. Compared with patients without LV-VTs, those with LV-VTs showed a significantly lower ablation efficacy (complete and partial success rates, 60 vs. 95%, *P* = 0.012; *Figure 4*). One patient in the RV group had RV perforation during ablation and got effective relief after pericardial drainage. There were no major complications associated with the procedure in other patients.

### Long-term follow-up after ablation

#### Ventricular tachycardia recurrence

**Figure 5:**
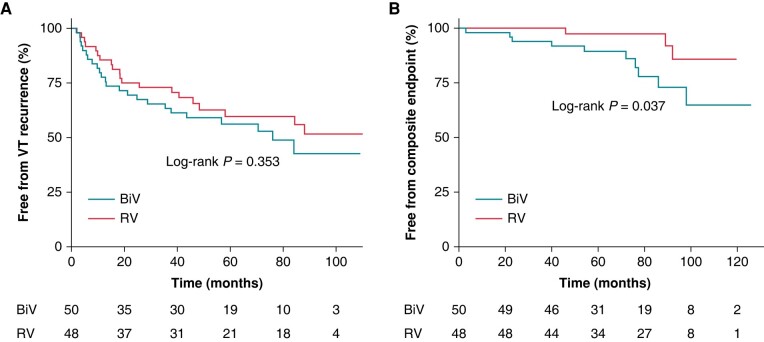
Kaplan–Meier plots illustrating survival rates free from clinical endpoints in patients who underwent VT ablation. (*A*) Survival free from VT recurrence. (*B*) Survival free from composite endpoint (all-cause death or heart transplantation). BiV, biventricular; RV, right ventricular; VT, ventricular tachycardia.

After a mean follow-up of 51 ± 34 months [median, 48 (22–83) months], cumulative VT-free survival was 52% (26/50) in the BiV group and 58% (28/48) in the RV group, respectively. The median time from ablation to this endpoint was 17 (7–39) months [13 (6–36) months in the BiV group vs. 18 (10–42) months in the RV group; *P* = 0.448]. The Kaplan–Meier curve showed no significant difference in VT-free survival between the two groups (log-rank *P* = 0.353, *Figure 5A*). Fifteen patients (15/50, 30%) in the BiV group and 13 (13/48, 27%) in the RV group had an ICD at the last follow-up. Among these patients, five from the BiV group (5/15, 30%) and two from the RV group (2/13, 15%) had appropriate ICD interventions (*P* = 0.512). Of 10 patients with LV-VTs in the BiV group, 5 (5/10, 50%) had VT recurrence at an average of 12 ± 7 months [median, 11 (8–13) months]. Among the remaining 40 patients without LV-VTs, 19 (19/40, 48%) had VT recurrence at 28 ± 27 months [median, 18 (6–41) months] after ablation. There was no difference in VT-free survival between these two subgroups (log-rank *P* = 0.887).

Univariate analysis showed that younger age, lower RVEF, and partial success or failure in the procedure were correlated with VT recurrence in the BiV group, which were adjusted by multivariate analysis (all *P* < 0.05; *Table 4*). Notably, LVEF [hazard ratio (HR) 0.96, 95% confidence interval (CI) 0.92–1.05; *P* = 0.148] or the presence of LV-VTs (HR 2.53, 95% CI 0.62–7.14; *P* = 0.472) was not associated with VT recurrence.

**Table 4:** Predictors of VT recurrence after ablation in patients with ARVC and BiV involvement

	Univariate analysis		Multivariate analysis	
	HR (95% CI)	*P*-value	HR (95% CI)	*P*-value
Age, years	0.96 (0.94–0.99)	0.012	0.96 (0.93–1.00)	0.041
Male sex, *n*	2.39 (0.31–18.38)	0.401		
Induced VT types, *n*	1.23 (0.93–1.62)	0.152		
With LV-VTs, *n*	1.70 (0.70–4.12)	0.239		
RVEF, %	0.89 (0.77–0.94)	0.001	0.93 (0.86–0.98)	0.015
LVEF, %	0.95 (0.90–1.01)	0.079	0.96 (0.92–1.05)	0.148
Acute complete success, *n*	0.18 (0.08–0.42)	<0.001	0.18 (0.07–0.45)	<0.001

ARVC, arrhythmogenic right ventricular cardiomyopathy; BiV, biventricular; CI, confidence interval; HR, hazard ratio; LV, left ventricular; LVEF, LV ejection fraction; RVEF, right ventricular ejection fraction; VT, ventricular tachycardia.

#### All-cause mortality and heart transplantation

A total of seven patients in the BiV group and two in the RV group died during follow-up at an average of 55 ± 34 months [median, 54 (23–86) months]. The cause of death was decompensated heart failure in six patients; two patients died of SCD (3 and 23 months after ablation, respectively), and one died of gastric cancer. Three patients in the BiV group and one in the RV group received heart transplantation because of refractory heart failure. The Kaplan–Meier curve showed a significantly lower composite endpoint-free survival in the BiV group (log-rank *P* = 0.037; *Figure 5B*).

## Discussion

### Main findings

To the best of our knowledge, this is the first study to compare the electrophysiological features and ablation outcomes between ARVC patients with BiV involvement and isolated RV involvement. The main findings are as follows: (i) BiV involvement correlated with fast clinical VTs, a high VT inducibility, and extensive arrhythmogenic substrates; (ii) LV-VTs were observed in 20% of the BiV group patients and associated with significantly reduced LVEF; (iii) catheter ablation achieved similar acute and long-term efficacy for VT control between these two groups, whereas the presence of LV-VTs increased acute ablation failure; and (4) younger age, lower RVEF, and non-acute complete success in the procedure were predictors of VT recurrence after ablation in patients with BiV involvement.

### Clinical characteristics of arrhythmogenic right ventricular cardiomyopathy patients with biventricular involvement and sustained ventricular tachycardias

Biventricular involvement has been reported in ARVC heart samples and CMR results and was associated with an increased risk of VAs.^13,19^ In a multi-centre study, 76% of patients with a pathologic diagnosis of ARVC had BiV involvement, of whom 75% documented clinical VAs.^11^ Sen-Chowdhry *et al*.^10^ found that BiV involvement increased 10-fold excess risk of non-sustained VTs. In another study on ablation for ARVC electrical storm, the enrolled patients had significant RV electrical and structural abnormalities, of whom 35% had LV dysfunction.^7^ Similarly, our present study revealed that half of the patients undergoing VT ablation displayed BiV involvement. Consistent with the CMR results, EPS revealed much larger RV electroanatomic scar areas in the BiV group. In ‘classic’ ARVC, low-voltage areas predominantly located in the perivalvular regions (peritricuspid and RV infundibulum) typically extending from the valve annuli to areas of the RV anterior and inferior free walls and were closely associated with VAs.^20^ Our study found that VTs in the BiV group also predominated from the RV, and the distributed pattern did not differ from that in the RV group, predominantly located in the subtricuspid area and the RVOT. Notably, these BiV group patients showed faster clinical VTs and a higher VT inducibility. Patients with BiV involvement had more remarkable RV and LV systolic dysfunctions, suggesting a potentially more significant impact on haemodynamics if the sustained VT episodes, which may partially explain the higher proportion of syncope history in this population. These study results indicate that BiV involvement likely implies worse arrhythmogenic substrates and arrhythmic events.

### Impact of biventricular involvement on left-sided ventricular tachycardias

Although BiV involvement increases the risk of heart failure and heart transplantation, its impact on LV arrhythmogenic substrates has not been well elucidated. In previous reports, RBBB-like VT was observed in 7.5–17% of ARVC patients and considered a manifestation of additional or predominant LV involvement in the disease. In Laredo *et al*.’s^21^ study, all RBBB-like VTs demonstrated an LV origin. However, Marchlinski *et al.*^22^ stated that up to half of such cases originated from the dilated inferior RV rather than the LV. Our present study observed and reproduced RBBB-like clinical VTs in 20% of the BiV group patients. In keeping with Laredo *et al.*’s^21^ result, we also confirmed an LV origin in all cases. These LV-VTs were predominated from the inferior and lateral LV walls and showed a much shorter CL and a significantly lower LVEF. Additionally, half of the cases were complicated with unstable haemodynamics during VT episodes. These results indicate that BiV involvement may result in multiple VA origins and increase the risk of BiV arrhythmias. Patients with significantly reduced LVEF appeared to have a higher risk of LV-VTs. For these patients, more aggressive interventions were strongly recommended.

### Ablation outcomes in arrhythmogenic right ventricular cardiomyopathy patients with biventricular involvement

Previous data reported that endo-epicardial combined ablation yielded a higher VT-free survival, while recent evidence indicated that the endocardial-alone approach could also be effective in a considerable number of patients.^16,23,24^ In Laredo *et al*.’s^7^ study, 83% of patients received endocardial ablation for ARVC electrical storm, and 85% of cases achieved freedom from electrical storm recurrence. Indeed, with the progression of replacement of normal myocardium and ventricular dilatation, ventricular wall thickness in ARVC decreased accordingly.^25^ Modern open-irrigated ablation catheters can achieve ablation lesions with a depth of 7–8 mm, theoretically enabling a transmural lesion from the endocardial surface, especially in the RV.^26,27^ In the present study, most patients only received endocardial ablation but effectively eliminated the clinical VTs at the end of the procedures. As most VTs in the BiV group patients originated from the RV, the endocardial ablation strategy achieved similar acute and long-term ablation efficacies as those in the RV group.

It is worth noting that nearly half of the patients in both groups experienced VT recurrence during follow-up, with the vast majority occurring within 20 months after ablation. This proportion seemed higher than that reported by previous literature, in which many more patients underwent the endocardial plus adjuvant epicardial ablation. We speculated that sufficient endocardial ablation can indeed eliminate the VT critical sites and subendocardial abnormal potentials. However, the undetected epicardial substrates may serve as an important contributor to VT recurrence. Moreover, we observed that 70% of LV-VT patients remained VT inducible after extensive endocardial ablation. However, among the three patients undergoing endo-epicardial ablation, all clinical VTs were completely terminated. Considering the thickness of the LV wall and the unique distribution pattern of arrhythmogenic substrates proceeding from the epicardium to the endocardium, epicardial mapping and ablation may be indispensable for LV-VAs in patients with ARVC. Similarly, in LV-dominant arrhythmogenic cardiomyopathy, such as desmoplakin cardiomyopathy, as most VT circuits were epicardial in LV, ablation procedures often required extensive interventions in the epicardium. Interestingly, desmoplakin cardiomyopathy could also present with BiV involvement phenotype. Two recent studies reported that 14–31% of patients with desmoplakin variants and VTs had mild or paralleled RV abnormalities, and nearly 30% of individuals displayed BiV VTs.^28,29^ Although these patients received adequate ablation in both ventricles and the epicardium, the long-term outcomes were unfavourable, and more than half of patients experienced VAs recurrence. In contrast, our study population was inclined to have predominant RV involvement with mild or moderate LV alterations, and the LV-originated VAs were not frequently observed. These discrepancies may partially explain the difference in ablation strategy and outcomes between these two populations.

### Identified predictors of ventricular tachycardia recurrence in the biventricular group patients

In this study, the clinical variables associated with VT recurrence after ablation in the BiV group were age, RVEF, and acute ablation efficacy. After multivariable analysis, younger age at presentation, lower RVEF, and partial success or failure in the procedure remained the independent risk factors.

Although several previous data reported VT recurrent predictors after ablation, the study results were inconsistent. In Berruezo *et al*.’s^30^ report, RV systolic function and acute ablation efficacy had no impact on VT recurrence, and the only related risk factor was the presence of predominant LV involvement. In another cohort study, Souissi *et al*.^31^ reported that RV dysfunction was associated with better long-term clinical response to ablation procedures, contrary to what one would expect. They gave a plausible explanation that the enlarged RV cavities may facilitate the contact and stability of radiofrequency. Our study is the first to investigate the predictors of VT recurrence in patients with BiV involvement. Interestingly, our results revealed that reduced RVEF increased VT recurrence. This could have been because more severe RV dysfunction usually suggests more significant RV structural alterations, thereby increasing the difficulty of complete elimination of potential arrhythmogenic substrates. In addition, lower RVEF itself has been reported to increase the risk of VAs in ARVC patients.^32^ Gasperetti *et al.*^33^ reported that programmed ventricular stimulation (PVS) is a useful risk-stratification tool for the primary prevention of ARVC cohort, and the inducible VTs during PVS indicated a four-fold risk of sustained VAs during follow-up. However, in our ablated cohort, neither the VT inducibility nor the induced VT types correlated with the VT recurrence. This result was also similar to that reported by previous studies.^24,30^

In another two retrospective studies, investigators analysed the relationships between acute ablation efficacy and long-term outcomes.^24,34^ In line with our results, they also demonstrated the predictive value of acute complete ablation success on VT recurrence. Compared with the patients with partial success and acute ablation failure, those who achieved complete success in our present study demonstrated an 82% decrease in VT recurrence. Young age is considered an independent risk factor of VAs in ARVC,^35,36^ and its predictive value in the ablation cohort remains controversial. Müssigbrodt *et al.*^24^ stated that younger age was associated with long-term freedom from VT recurrence. However, this result was summarized from the univariate analysis. Other studies did not report any significant difference in age between patients with and without VT recurrence.^30,34^ We investigated this relationship in the subgroup of BiV involvement and found that younger age at presentation independently predicted VT recurrence after ablation. Although this finding requires confirmation in a larger cohort, our result, to some extent, implied that age, a simple and easily accessible clinical variable, can provide considerable prognostic information for risk-stratification purposes when evaluating ablation outcomes in ARVC patients with BiV involvement.

### Study limitations

There are several potential limitations to note. This was a retrospective observational study including patients with ARVC and documented sustained VT with available CMR data, and therefore, selection bias could not be avoided. Moreover, long-term VT-free survival may have been affected by a degree of ascertainment bias. As the ICD device is quite expensive in China and also associated with a series of device-related complications, only 30% of patients implanted, and others refused primarily due to financial reasons. Therefore, it was likely that asymptomatic VT episodes occurring outside the ECG monitoring window during follow-up were not captured. Furthermore, only a limited number of patients underwent epicardial mapping and ablation because of the fear of procedural complications associated with epicardial puncture and ablation. The additional use of this therapeutic strategy may further improve the acute complete success rate and long-term ablation efficacy, especially for patients with LV-VTs. Finally, despite the large study sample size for such a rare disease and for the complex interventions, the size was still small to account for various confounders. These limitations should be taken into consideration when interpreting our results.

## Conclusions

Biventricular involvement in ARVC correlated with a severe arrhythmic phenotype and increased the risk of left-sided VTs. Catheter ablation maintained its efficacy for VT control in these patients, whereas the presence of LV-VTs increased acute ablation failure. Young age, reduced RVEF, and non-acute complete success in the procedure independently predicted VT recurrence after ablation in patients with BiV involvement.

## Supplementary Material

euae059_Supplementary_Data

## Data Availability

The data that support the findings of this study are available from the corresponding author upon reasonable request.
