# Supplementary material for: Catheter ablation of ventricular tachycardia in patients with arrhythmogenic right ventricular cardiomyopathy and biventricular involvement
Source: Europace. 2024 Feb 28;26(3):euae059. doi: 10.1093/europace/euae059 (PMC10946245; doi:10.1093/europace/euae059)

**Supplementary Material**

Lishui Shen, Shangyu Liu, Zhenhao Zhang, et al. Catheter Ablation of Ventricular Tachycardia in Patients with Arrhythmogenic Right Ventricular Cardiomyopathy and Biventricular Involvement*. Europace*

**Table S1.** Mutations represented in the study population.

**Table S2.** Procedural data of 10 cases with acute ablation failure.

**Table S3.** Procedural data of 10 patients inducing LV-VTs in BiV group.

**Figure S1.** ECG and CMR findings of a representative patient with ARVC and BiV involvement.

**Figure S2**. Representative examples of ECGs in patients with BiV involvement and isolated RV involvement.

| **Gene; n (%)** | **Nucleotide Change** | **Amino Acid Change** | **Variant Type** | **Patients, n** |
| --- | --- | --- | --- | --- |
| ***PKP2*; 33 (38)** | c.224-3C>G | Splice | Splice | 1 |
|  | c.235C>T | Arg79* | Nonsense | 2 |
|  | c.336+1G>A | Splice | Splice | 1 |
|  | c.498C>G | Tyr166* | Nonsense | 1 |
|  | c.508C>T | Gln170* | Nonsense | 1 |
|  | c.568delG | Val190fs | Frameshift | 1 |
|  | c.731_744del | Pro244Glnfs | Frameshift | 1 |
|  | c.746G>C | Ser249Thr | Missense | 1 |
|  | c.801delT | Thr267fs | Frameshift | 1 |
|  | c.870G>A | Trp290* | Nonsense | 2 |
|  | c.968_969del | Gln323Argfs | Frameshift | 2 |
|  | c.1063C>T | Arg355* | Nonsense | 1 |
|  | c.1170+1G>A | Splice | Splice | 1 |
|  | c.1211_1212insT | Ser406fs | Frameshift | 1 |
|  | c.1271 T>C | Phe 424Ser | Missense | 2 |
|  | c. 1759delG | Ser587fs | Frameshift | 2 |
|  | c. 1849C>T | Gln617* | Nonsense | 1 |
|  | c.1891G>A | Glu631Lys | Missense | 1 |
|  | c.1951C>T | Arg651* | Nonsense | 2 |
|  | c.1978C>T | Gln660* | Nonsense | 3 |
|  | c.1994C>G | Pro665Arg | Missense | 1 |
|  | c. 2359C>T | Leu787Phe | Missense | 1 |
|  | c.2509delA | Val837fs | Frameshift | 1 |
|  | c. 2544G>A | Trp848* | Nonsense | 1 |
|  | c.2554delG | Glu852fs | Frameshift | 1 |
| ***DSG2*; 9 (10)** | c.146G>A | Arg49His | Missense | 3 |
|  | c.685A>G | Arg229Gly | Missense | 1 |
|  | c.874C>T | Arg292Cys | Missense | 2 |
|  | c.1334C>T | Ser445Phe | Missense | 1 |
|  | c.1520G>A | Cys507Tyr | Missense | 2 |
| ***DSC2*; 4 (5)** | c.229G>A | Gly77Ser | Missense | 1 |
|  | c.287T>C | Ile96Thr | Missense | 1 |
|  | c. 942+3A>G | Splice | Splice | 1 |
|  | c.1276G>A | Glu426Lys | Missense | 1 |
| ***DSP*; 4 (5)** | c.621G>T | Trp207Cys | Missense | 1 |
|  | c. 3337C>T | Arg1113 | Nonsense | 1 |
|  | c. 4043T>G | Leu1348Arg | Missense | 1 |
|  | c.6634G>T | Gly2212Ter | Missense | 1 |
| ***TMEM43*; 4 (5)** | c.332C>T | Pro111Leu | Missense | 1 |
|  | c.1073C>T | Ser358Leu | Missense | 3 |
| ***LMNA*; 2 (2)** | c.725C>T | Ala242Val | Missense | 2 |
| ***PLN*; 2 (2)** | c.40_42delAGA | Arg14del | Deletion | 2 |
| ***DES*; 1 (1)** | c.1255C>T | Pro419Ser | Missense | 1 |

ACMG = American College of Medical Genetics and Genomics; DES = desmin; DSC2 = desmocollin-2; DSG2 = desmoglein-2; DSP = desmoplakin; LMNA = lamin A/C; PKP2 = Plakophilin-2; PLN = phospholamban; TMEM43 = transmembrane protein 43.

**Table S1.** Mutations represented in the study population.

**Table S2. Procedural data of 10 cases with acute ablation failure**

| **Patient number** | **Induced BiV-VTs** | **Induced only RV-VTs** | **No. of induced VT types** | **ENDO mapping** | **EPI mapping** | **ABL failure of clinical LV-VTs** | **ABL failure of clinical RV-VTs** | **Hemodynamic instability or VF** | **Complication** |
| --- | --- | --- | --- | --- | --- | --- | --- | --- | --- |
| #3 | No | Yes | 3 | Yes | No | - | Yes | Yes | No |
| #11 | Yes | No | 3 | Yes | No | Yes | No | No | No |
| *23 | No | Yes | 1 | Yes | No | - | Yes | No | No |
| #45 | Yes | No | 6 | Yes | No | Yes | Yes | Yes | No |
| *49 | No | Yes | 3 | Yes | Yes | - | Yes | No | RV perforation |
| #62 | Yes | No | 4 | Yes | No | Yes | No | Yes | No |
| *66 | No | Yes | 3 | Yes | No | - | Yes | Yes | No |
| #72 | No | Yes | 2 | Yes | No | - | Yes | No | No |
| *89 | No | Yes | 4 | Yes | No | - | Yes | No | No |
| #93 | Yes | No | 4 | Yes | No | Yes | Yes | Yes | No |

ABL, ablation; BiV, biventricular; ENDO, endocardial; EPI, epicardial; LV, left ventricular; RV, right ventricular; VF, ventricular fibrillation; VT, ventricular tachycardia

# indicating the patients from the BiV group, and * indicating the patients from the RV group

**Table S3. Procedural data of 10 patients inducing LV-VTs in BiV group**

| **Patient number** | **Clinical LV-VT CL (ms)** | **No. of induced LV-VTs** | **Hemodynamics during LV-VTs** | **Clinical RV-VT CL (ms)** | **No. of induced RV-VTs** | **Hemodynamics during RV-VTs** | **ABL strategy** | **Acute ABL efficacy** | **Complication** |
| --- | --- | --- | --- | --- | --- | --- | --- | --- | --- |
| 7 | 270 | 1 | Stable | 300 | 2 | Stable | ENDO | CS | No |
| 11 | 330 | 1 | Stable | 300 | 2 | Stable | ENDO | Fail | No |
| 13 | 260 | 2 | Stable | 0 | 0 | NA | ENDO + EPI | CS | No |
| 19 | 330 | 1 | Stable | 230 | 2 | VF | ENDO | PS | No |
| 28 | 250 | 2 | VF | 330 | 1 | Stable | ENDO + EPI | PS | No |
| 45 | 260 | 3 | VF | 310, 360 | 3 | Stable | ENDO | Fail | No |
| 62 | 290 | 1 | Unstable | 360 | 3 | Stable | ENDO | Fail | No |
| 64 | 240 | 2 | Unstable | 0 | 0 | NA | ENDO | PS | No |
| 81 | 300 | 1 | Stable | 420 | 1 | Stable | ENDO + EPI | CS | No |
| 93 | 250 | 1 | Unstable | 330 | 3 | Stable | ENDO | Fail | No |

ABL, ablation; CL, cycle length; CS, complete success; ENDO, endocardial; EPI, epicardial; LV, left ventricular; PS, partial success; RV, right ventricular; VF, ventricular fibrillation; VT, ventricular tachycardia

**Figure S1**. ECG and CMR findings of a representative patient with ARVC and BiV involvement.

**-A**, Basal ECG showing epsilon waves in precordial leads, flattened T-waves in inferior leads, and inverted T-waves in anterior and lateral leads.

**-B**, End-diastolic frame of cine CMR sequence in long-axis showing significantly enlarged right ventricle and right atrium.

**-C** and **D**, Postcontrast image in long-axis and short-axis showing extensive myocardial fibrosis in the form of stria of late gadolinium enhancement in the endo-epicardium of the right ventricular free wall, epicardium of the left ventricular free wall and the endocardial layer of the interventricular septum (arrows).


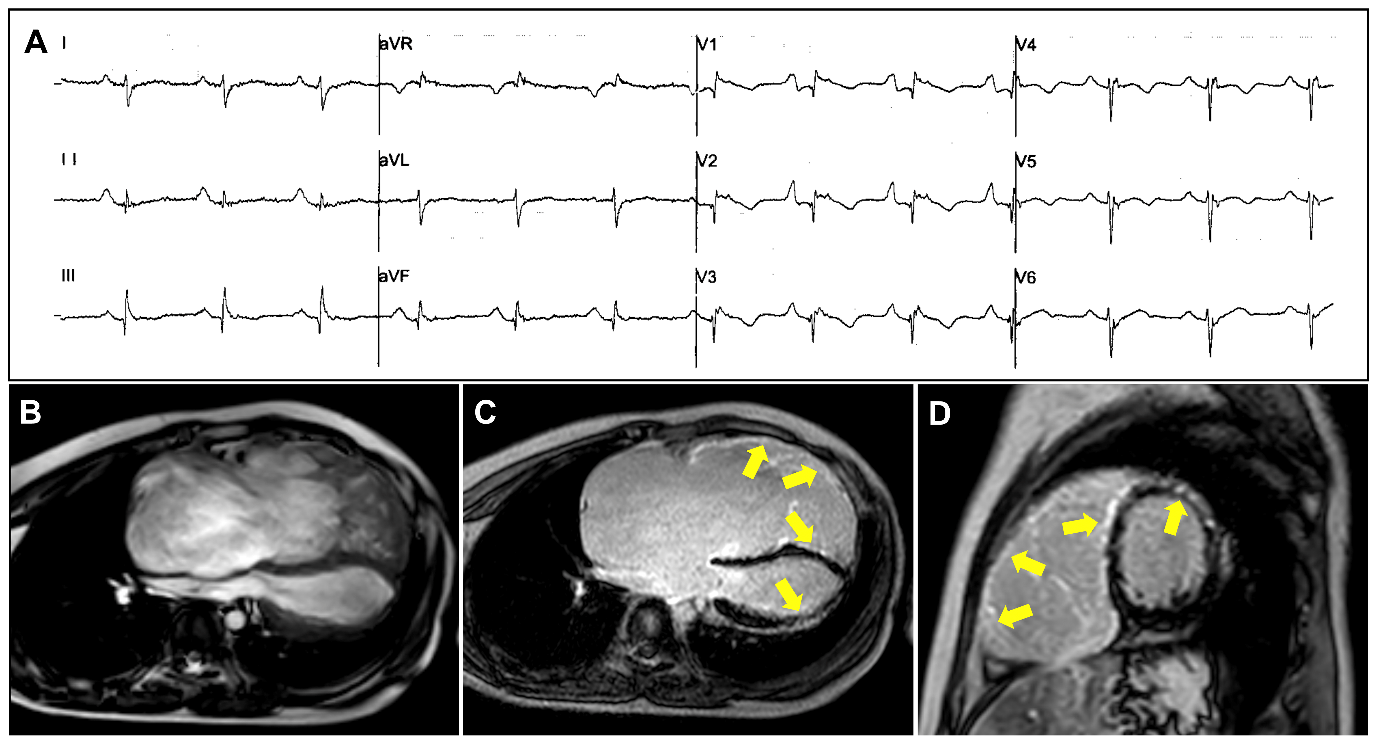


**Figure S2**. Representative examples of ECGs in patients with BiV involvement and isolated RV involvement.

**-A**, ECG from a patient with isolated RV involvement showing T-wave inversion in leads V1 to V3 and normal QRS voltage in chest leads and limb leads.

-**B**, ECG from a patient with BiV involvement showing T-wave inversion in leads V1 to V6 and low QRS voltage in limb leads.


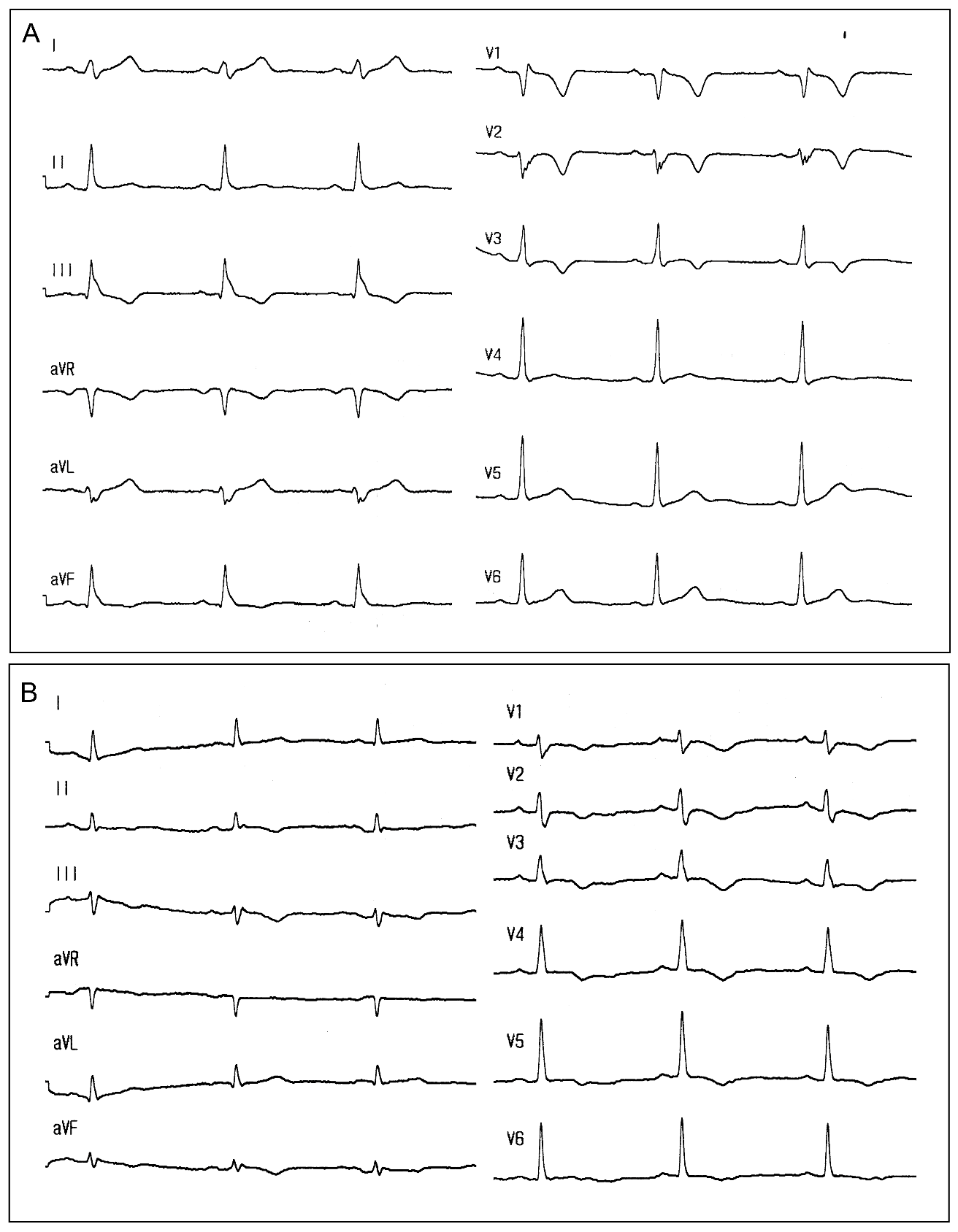

Supplement: euae059_Supplementary_Data [file euae059_supplementary_data.docx]
